# Primary Bone Lymphoplasmacytic Lymphoma Presenting with Spinal Cord Compression: A Case Report

**DOI:** 10.4274/Tjh.2012.0072

**Published:** 2013-12-05

**Authors:** Yang Lei, Liu Zi, Su Long, Li Pei, Li Wei

**Affiliations:** 1 Jilin University, Department of Hematology and Oncology, Changchun, China

**Keywords:** Primary bone lymphoma, Lymphoplasmacytic lymphoma, Spinal cord compression

## Abstract

Primary bone lymphoma is a rare disease, and the main pathological type is diffuse large B-cell lymphoma. The occurrence of follicular, marginal zone and lymphoplasmacytic lymphomas is rare. Vertebras are also sites that can be affected, and spinal cord compression is reported in 14% of patients with vertebral involvement. However, there is no report on primary vertebral lymphoplasmacytic lymphoma with spinal cord compression. The present report presents one case of primary vertebral lymphoplasmacytic lymphoma with spinal cord compression and increased serum and urine λ light chain, without an elevated heavy chain of immunoglobulin.

**Conflict of interest:**None declared.

## Abstract (tr)

Primer kemik lenfoması nadir bir hastalık olup, en sık patolojik tipi difüz büyük B hücreli lenfomadır. Folliküler, marjinal lenfoma ya da lenfoplazmasitik lenfoma alt tipleri nadirdir. Vertebra tutulan bölgelerden biridir ve spinal kord basısı vertebral tutulumu olan hastaların %14’ünde bildirilmiştir. Bununla birlikte, spinal kord basısı ile başvuran bir primer vertebra lenfoplazmasitik lenfoma daha önce rapor edilmemiştir. Bu yazıda spinal kord kompresyonuna neden olmuş primer vertebra lenfoplazmasitik lenfoması olan ve serum, idrar λ hafif zincir miktarı artmış ancak immünglobulin ağır zinciri normal bulunan bir olgu sunulmuştur.

## INTRODUCTION

Primary bone lymphoma (PBL) is defined as lymphoma localized to the bone without evidence of involvement of lymph nodes or other tissues at presentation. It one of the rarest primary bone malignancies, accounting for less than 5% of all primary bone tumors [1]. PBL constitutes less than 1-2% of all malignant lymphomas in adults [2]. Most PBLs are primary bone diffuse large B-cell type lymphomas with a rare occurrence of follicular, marginal zone and lymphoplasmacytic types [3]. The long bones are primarily affected and the femur is the most commonly involved location as a single site [2,4]. The common signs and symptoms are local bone pain with or without soft tissue swelling and pathological fracture. Spinal cord compression is reported in 14% of patients with vertebral involvement but the presence of B symptoms is relatively uncommon [2,5]. PBL has a better prognosis following radiotherapy and chemotherapy than many other malignant tumors, and therefore early identification allows for appropriate treatments [2,6]. In this report, the authors present a 61-year-old patient with a primary vertebra lymphoplasmacytic lymphoma presenting with spinal cord compression. 

## CASE REPORT

A 61-year-old woman presented to the emergency department with a 3-month history of progressive chest and back pain, 1-month history of numbness and weakness of the lower extremities, and paraplegia for 1 day. Initially, the patient had a paroxysmal pain of the chest and lower back, which spread progressively to the bilateral scapula, oxter, and praecordia. Two months later, she felt numbness in her left lower extremity. After 1 week, she felt weakness in the lower extremities and had difficulty in walking. In three months, the symptoms worsened and hypoesthesia appeared. She became paraplegic the day before admission to hospital. The history revealed no cardiac, bowel, or bladder problems and her pain was not associated with motor or sensory neurological deficits at presentation. On physical examination she had no superficial lymphadenopathy or hepatosplenomegaly. The superficial hypoesthesia was located below the bilateral papilla plane. Tenderness and pain in percussion were positive at the level of vertebra T1-T3, in addition to a mild pain at the level of vertebra L4. Muscular force was normal for upper limbs, and the forces of hip, knee, and ankle joints were Grade III for both extending and flexing. Patellar reflex was strengthened and Achilles tendon reflex was normal. Computerized tomography (CT) scan showed a space-occupying lesion located in and outside the left canalis spinalis and foramen intervertebral levels T1-T3, accompanied with the destruction of the second vertebra. Serum calcium, albumin, and lactate dehydrogenase were within normal range. β2-microglobulin was slightly increased (2.57 mg/L; normal range is 0.7-1.8 mg/L). Blood and urine immunofixation were positive for λ chain. Serum-free lambda light chain was 175.3 mg/L (normal range is 6.72-22.81 mg/L), and κ chain was 10.6 mg/L (normal range is 5.81-21.04 mg/L). The 24-h urine λ chain was 949.2 mg (normal range is <7.8 mg). Serum IgG was normal, but a slight decrease was seen for IgA and IgM, at 0.616 g/L (normal range is 0.7-4.0 g/L) and 0.192 g/L (normal range is 0.4-2.3 g/L), respectively. The erythrocyte sedimentation rate was 21 mm/h (normal range is 0-20 mm/L for females), and the International Prognostic Index score was 4. Bone marrow examinations with both smear and biopsy were normal. Informed consent was obtained from the patient. 

**Figure 1:**
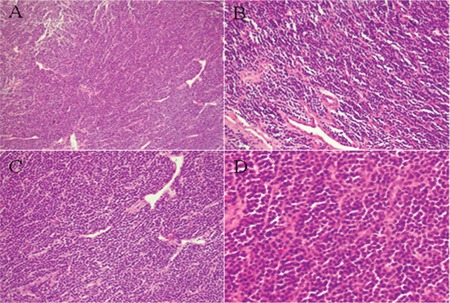
The hematoxylin-eosin (H&E) stained laminectomy material revealed middle-sized tumor cells with plasma cell features that were characterized by abundant cytoplasm and asymmetrical nuclei. The morphology suggested a proliferative disease of plasma cells

**Figure 2:**
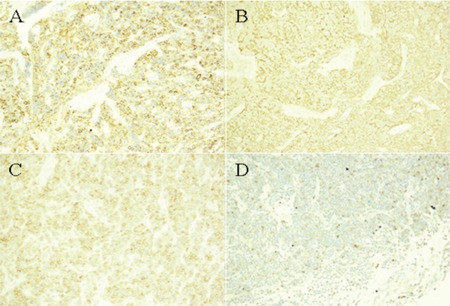
The immunohistochemical staining resulted in CD20 (+) (Figure 2A), CD138 (±) (Figure 2B), CD56 (+) (Figure 2C), CD79a (+) (Figure 2D) cells. The pathological diagnosis was lymphoplasmacytic lymphoma

On 30 September 2010, the patient received decompressive laminectomy, and the excision material was evaluated by hematoxylin-eosin (H&E) staining and immunohistochemistry. The results revealed diffuse tumor cell proliferation, which infiltrated and damaged the adjacent bone and soft tissues. The middle-sized tumor cells showed plasma cell features that were characterized by abundant cytoplasm and asymmetrical nuclei. The chromatin was granulated and Russell bodies could be observed. No plasmablasts could be found. The morphology suggested a proliferative disease of plasma cells (Figures 1A-1D). The immunohistochemical staining result was CD20 (+) (Figure 2A), CD138 (±) (Figure 2B), CD56 (+) (Figure 2C), CD79a (+) (Figure 2D), CD38 (+), Lambda (+), NUM-1 (+), Ki-67 (some +), PAX5 (weakly positive), Bcl-2 (+/-), CyclinD1 (-), CD21 (-), CD5 (-), CD3 (-), CK-P (-), CD43 (-), CD23 (-), CD10 (-), Bcl-6 (-), MPO (-), Kappa (-), EMA (-), and EBER (-). The pathological diagnosis was lymphoplasmacytic lymphoma. 

**Figure 3:**
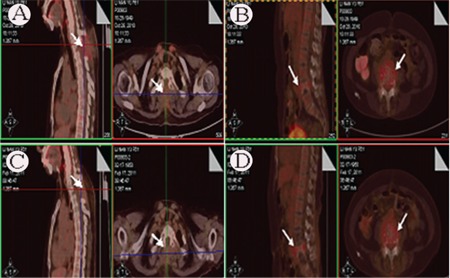
PET-CT of the patient revealed FDG uptake at second thoracic (Figure 3A, C) and fourth lumbar vertabrae (Figure 3B, D).

Positron emission tomography (PET)-CT was utilized to stage and determine the disease focus. The results revealed that the second vertebra had hypermetabolism accompanied with bone destruction, and the standardized uptake value (SUV) was 3.2 (average: 2.1) (Figure 3A). The fourth lumbar vertebra was also hypermetabolic accompanied with bone destruction, and the SUV was 3.0 (Figure 3B), which suggested infiltration of lymphoma and compression fracture of the lumbar vertebra. There was no significant hypermetabolic focus in other sites. 

Local radiation therapy was given to the second thoracic and fourth lumbar vertebras (total: 44 Gy/22 f). The back pain gradually disappeared and muscle strength and feeling gradually recovered during the treatment. The patient then performed functional training for 1 month. Muscular tension and strength recovered to normal and she could move freely. Reexamination of the serum and urine immunofixation showed positivity of the λ chain. The λ chain was 413.1 mg (decrease of >50%) for 24 h. PET-CT showed significantly decreased focus in the second thoracic and fourth lumbar vertebras (Figures 3C and 3D). After that, the patient received R-CHOP chemotherapy (R: rituximab, 600 mg; C: cyclophosphamide, 1125 mg; H: epirubicin, 112.5 mg; O: vindesine, 4 mg; P: prednisone, 100 mg). 

## DISCUSSION

PBL is a rare presentation of non-Hodgkin’s lymphoma. It was first described as a distinct clinicopathological entity in 1939 by Parker and Jackson [7]. The real prevalence of PBL is unclear because of the considerable difficulty in distinguishing primary from secondary bone lymphoma [2]. According to Coley’s criteria [8], primary bone lymphoma should have: 1) a primary focus in a single bone; 2) positive histological diagnosis; and 3) no evidence of distant soft tissue or distant lymph node involvement. Regional lymph node involvement at diagnosis is not considered as an exclusion criterion [9,10]. Recently, multiple bone involvement was also recognized as primary bone lymphoma, as long as the other 2 criteria are met [9]. According to the WHO classification, lymphoma involving the bone can be classified in 4 groups: group 1, lymphoma in a single bone site with or without regional lymph node involvement; group 2, lymphoma in multiple bones, but no visceral or lymph node involvement; group 3, bone tumor with involvement of other visceral sites or lymph nodes at multiple sites; and group 4, lymphoma involving any other site and found by bone biopsy that was done to rule out possible involvement [2]. In this case, the patient was considered as having PBL involving 2 separate sites of the bone based on the result of accessory examination. 

The stage of PBL was established with the Ann Arbor staging system. Single localized bone lesions were classified as Stage IE, and in the case of lymph node involvement on the same side of the diaphragm, patients were considered to have Stage IIE. If lymph nodes in both sides of the diaphragm were involved, the case was considered as Stage IIIE. Stage IVE disease was defined as cases of multiple sites of bone involvement. Hence, the present patient was evaluated as a Stage IVE case. 

Lymphoplasmacytic lymphoma (LPL) is one of the rare subtypes accounting for only 1% to 2% of all non-Hodgkin’s lymphomas. This lymphoma arises from plasma cells, which are mature B-lymphocytes [11]. It is most often associated with increased IgM protein produced by the lymphoma cells and is commonly referred to as Waldenström’s macroglobulinemia (WM), which is considered an indolent lymphoma; the disease progresses very slowly and patients usually live many years after diagnosis. Recently, there was also a case report of non-secretory immunoglobulin of LPL [12]. Instead of the signs and symptoms caused by infiltration and the circulating IgM of WM, such as lymphadenopathy, hepatosplenomegaly or hyperviscosity, our patient only presented with increased λ chain and spinal cord compression. The immunohistochemistry revealed positive staining for CD20, CD79a, and Lambda of B cell markers and negative for CD38 and CD138 of plasma cells, and so this patient was diagnosed with lymphoplasmacytic lymphoma. It is difficult to rule out plasmacytoma when the bone is involved, but B cell antigen is negative in these patients. 

Several studies have suggested that combined modality (chemotherapy and radiotherapy) was the best treatment for patients with PBL [2,13]. Beal et al. concluded that PBL patients treated with combination chemotherapy and irradiation had significantly better survival than patients treated with a single modality (chemotherapy or radiotherapy alone), but the 5-year overall survival rate between the 2 groups was not significantly different [2,14]. Ramadan et al. reported patients with advanced-stage disease who received chemotherapy plus irradiation with a poor outcome when compared with those who received chemotherapy alone (10-year overall survival rates were 25% and 56%, respectively) [4]. However, this difference must be very cautiously interpreted because the decision to use radiotherapy was individualized. It is possible that patients with more biologically aggressive disease were more likely to receive irradiation, obscuring its impact [3]. Moreover, this result was derived from PBL of diffuse large-cell lymphoma [3]. However, there was no available information in this report about lymphoplasmacytic lymphoma.

After therapeutic and diagnostic surgery, the patient received radiation therapy for local lesions of the vertebras. As she was in an advanced stage (Stage IVE), the R-CHOP regimen was given for the subsequent treatment. She is currently alive after the treatment and was periodically reexamined for almost 1 year; there was no sign of disease progression or relapse. 

## CONFLICT OF INTEREST STATEMENT

The authors of this paper have no conflicts of interest, including specific financial interests, relationships, and/ or affiliations relevant to the subject matter or materials included.
